# Alginate Microsphere Encapsulation of Drug-Loaded Nanoparticles: A Novel Strategy for Intraperitoneal Drug Delivery

**DOI:** 10.3390/md20120744

**Published:** 2022-11-26

**Authors:** Karianne Giller Fleten, Astrid Hyldbakk, Caroline Einen, Sopisa Benjakul, Berit Løkensgard Strand, Catharina de Lange Davies, Ýrr Mørch, Kjersti Flatmark

**Affiliations:** 1 Department of Tumor Biology, Institute for Cancer Research, Norwegian Radium Hospital, Oslo University Hospital, 0379 Oslo, Norway; 2 Faculty of Medicine, Institute of Clinical Medicine, University of Oslo, 0372 Oslo, Norway; 3 Department of Biotechnology and Nanomedicine, SINTEF Industry, 7034 Trondheim, Norway; 4 Department of Physics, Norwegian University of Science and Technology (NTNU), 7034 Trondheim, Norway; 5 Department of Biotechnology and Food Science, Norwegian University of Science and Technology (NTNU), 7034 Trondheim, Norway; 6 Department of Gastroenterological Surgery, The Norwegian Radium Hospital, Oslo University Hospital, 0379 Oslo, Norway

**Keywords:** alginate, microspheres, nanoparticles, poly(alkyl cyanoacrylate), cabazitaxel, peritoneal metastasis

## Abstract

Alginate hydrogels have been broadly investigated for use in medical applications due to their biocompatibility and the possibility to encapsulate cells, proteins, and drugs. In the treatment of peritoneal metastasis, rapid drug clearance from the peritoneal cavity is a major challenge. Aiming to delay drug absorption and reduce toxic side effects, cabazitaxel (CAB)-loaded poly(alkyl cyanoacrylate) (PACA) nanoparticles were encapsulated in alginate microspheres. The PACAlg alginate microspheres were synthesized by electrostatic droplet generation and the physicochemical properties, stability, drug release kinetics, and mesothelial cytotoxicity were analyzed before biodistribution and therapeutic efficacy were studied in mice. The 450 µm microspheres were stable at in vivo conditions for at least 21 days after intraperitoneal implantation in mice, and distributed evenly throughout the peritoneal cavity without aggregation or adhesion. The nanoparticles were stably retained in the alginate microspheres, and nanoparticle toxicity to mesothelial cells was reduced, while the therapeutic efficacy of free CAB was maintained or improved in vivo. Altogether, this work presents the alginate encapsulation of drug-loaded nanoparticles as a promising novel strategy for the treatment of peritoneal metastasis that can improve the therapeutic ratio between toxicity and therapeutic efficacy.

## 1. Introduction

Microparticulate drug delivery systems offer numerous advantages in drug administration due to their structural and functional properties, including improved drug stability, controlled and sustained drug release, reduced drug toxicity, and target specificity [1]. Microparticles can be administrated by several routes (e.g., by oral ingestion, inhalation, topical use, or direct injection into the blood or specific tissues) and have been shown to have a strong therapeutic impact for many indications, including cancer, diabetes, cardiovascular diseases and neurological disorders [1,2]. The particle material is usually based on biocompatible polymers, where the alginate polysaccharide family is shown to be one of the biopolymers with the widest biomedical applicability [3]. Alginates are naturally occurring linear polymers derived from the cell walls of brown algae, that crosslink with divalent cations to form highly porous hydrogels with an inert aqueous environment within the gel matrix [4]. Due to their low toxicity and immunogenicity profile, alginate hydrogels have been broadly investigated for use in biomedical applications, including encapsulation of islet cells for diabetes type 1 treatment, wound healing, and cancer treatment [3,5,6,7].

The peritoneal cavity is a common metastatic site for malignancies of abdominopelvic organs, and is associated with a poor prognosis [8,9]. Cytoreductive surgery (CRS) followed by hyperthermic intraperitoneal chemotherapy (HIPEC) is the only curative treatment currently available for these patients. This treatment is, however, only suitable for a subgroup of patients and many of them experience a relapse—highlighting the need for new treatment options [10,11]. These patients respond poorly to systemic therapy, and intraperitoneal administration of cytotoxic drugs has therefore been used to achieve high local drug concentrations. The drugs are, however, rapidly cleared from the peritoneal cavity [12]. Hence, there is a need for therapeutic approaches that prolong the residence time of the drugs in the peritoneal cavity to increase drug exposure to peritoneal tumors [13]. This can be achieved by encapsulation of drugs into carriers, to both increase drug retention and reduce systemic toxicity. We have previously shown promising drug retention and improved treatment efficacy by encapsulating cabazitaxel (CAB) in poly(alkyl cyanoacrylate) (PACA) nanoparticles (NPs) [14]. CAB is a second-generation taxane approved for the treatment of patients with hormone-refractory metastatic prostate cancer [15]. This PACAB platform was shown to be a robust drug delivery system for treatment of peritoneal cancers, and is currently under commercial development. CAB has previously been nanoformulated in liposomes, lipospheres and PACA NPs for cancer treatment [16,17,18,19,20], but this was, to our knowledge, the first work presenting CAB-loaded NPs for treatment of intraperitoneal cancers. To potentially prolong drug retention in the peritoneal cavity, we hypothesized that encapsulation of the PACAB NPs into alginate microspheres would improve the balance between therapeutic efficacy and toxicity. 

**Figure 1:**
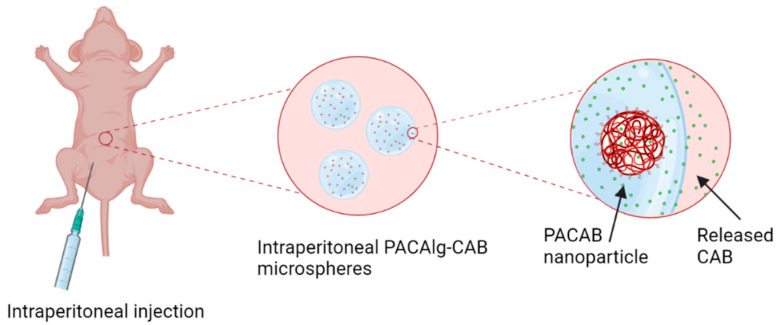
Graphical overview of the PACAlg-CAB drug delivery system. PACAlg-CAB microspheres are injected directly into the peritoneal cavity, where CAB is released.

In this study, we present the PACAlg technology, where alginate microparticles encapsulating PACAB NPs were designed and synthesized to improve intraperitoneal drug retention and reduce drug toxicity (Figure 1). The technology was evaluated by physicochemical characterization of the PACAlg microparticles, in vitro cytotoxicity studies, and finally, CAB biodistribution and treatment efficacy studies were conducted in patient-derived xenograft (PDX) models mimicking peritoneal metastasis. 

## 2. Results

### 2.1. Characterization of PACA Nanoparticles 

Empty PACA NPs had an average size of 168 nm, a polydispersity index (PDI) of 0.14, and a zeta potential of −4.3 mV. PACA NPs loaded with both CAB and the fluorescent dye NR668 were in the size range of 154–165 nm, had a PDI below 0.20, a zeta potential of −2.0 mV and a CAB concentration of 8.8–9.1 mg/mL (84–87% encapsulation efficiency). PACA NPs loaded with only CAB had a size of 124 nm, PDI of 0.26, a zeta potential of −2.8 mV and a CAB concentration of 9.1 mg/mL (87% encapsulation efficiency). NP surface PEGylation resulted in colloidal stability and no NP aggregation was observed in any of the batches.

### 2.2. Characterization of PACAlg Microspheres 

**Figure 2:**
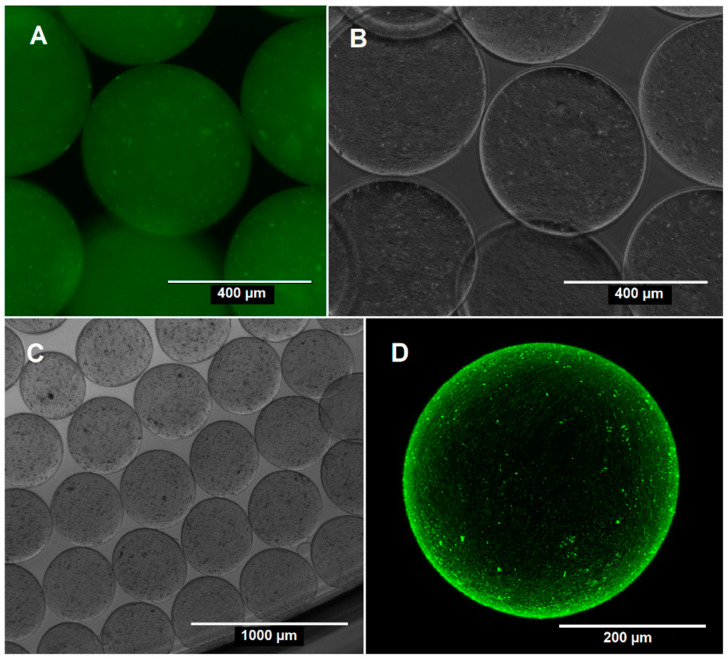
Representative micrographs of PACAlg microspheres encapsulating NR668/CAB-loaded PACA NPs. (**A**) Fluorescence micrograph; (**B**,**C**) Phase contrast micrographs; (**D**) Cross section of a microsphere imaged by confocal laser scanning microscopy (CLSM).

PACAlg microspheres were produced by an extrusion dripping technique based on the electrostatic pulling of extruded droplets, resulting in spherical hydrogels with an average diameter of 448 ± 32 µm (Figure 2). The relatively small standard deviation in microsphere diameter indicated a high monodispersity of the microspheres. Seventy percent of the PACAlg microspheres had an aspect ratio (AR) < 1.1 and a spherical factor (SF) < 0.05 and were thus stated as spherical. The remaining microsphere portion had AR and SF values below 1.54 and 0.23, respectively. Empty microspheres had a slightly smaller diameter of 334 ± 22 µm (data not shown). The PACA NPs within the PACAlg microspheres were distributed throughout the alginate hydrogel matrix, where the NP density was observed to gradually increase towards the hydrogel surface (Figure 2D). 

**Figure 3:**
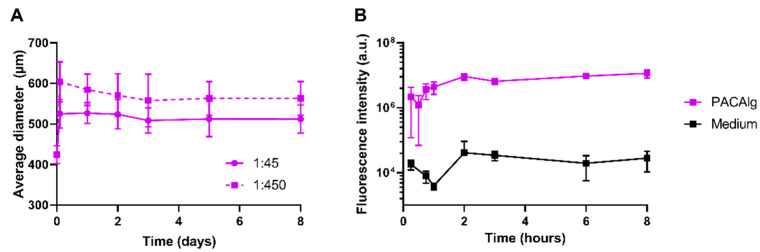
Stability of PACAlg microspheres. (**A**) Average diameters of PACAlg microspheres incubated in PBS (pH 7.4) at dilution factors of 1:45 and 1:450 volumetric ratios at 37 °C over an 8-day period. Data represent mean ± standard deviation (SD) (n = 20). (**B**) Fluorescence intensity detection of NR668-labeled PACA NPs encapsulated in PACAlg microspheres following 8-h incubation in PBS (pH 7.4) at a 1:450 dilution at 37 °C. Data represents mean ± SD (n = 3).

PACAlg size stability studies in physiological buffer (PBS, pH 7.4, 37 °C) showed microsphere swelling immediately after medium addition, with an increase in microsphere diameter of up to 650 µm (Figure 3A). The degree of swelling compared to the initial diameter rose as the dilution factor increased, as shown by a 25% and 44% swelling for dilution factors of 1:45 and 1:450, respectively (Figure 3A). After the initial swelling, the microspheres remained stable in a physiological buffer with regard to size and morphology for at least 8 days. 

NP retention within the PACAlg microspheres was studied by measuring the fluorescence intensity of PACA-NR668 NPs within the spheres and the surrounding incubation medium. Ninety-nine percent of the fluorescence intensity was retained within the microspheres for at least 8 h after incubation—indicating no leakage of fluorescent NPs or free fluorophore (Figure 3B). 

### 2.3. PACAlg Drug Release

**Figure 4:**
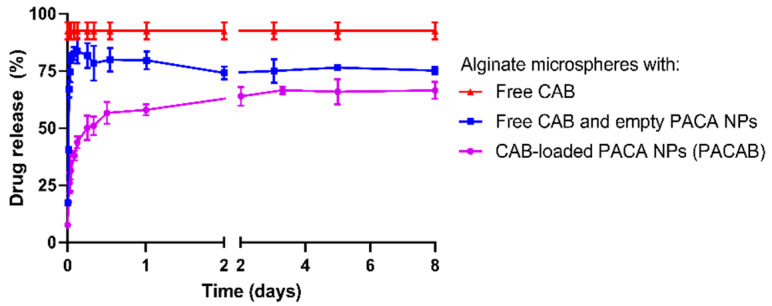
CAB drug release kinetics. Percentage CAB release from alginate microspheres with free CAB (red line), free CAB and empty PACA NPs (blue line) and PACAB NPs (purple line) after 8 days of microsphere incubation in PBS (pH 7.4) with 0.5% (*w*/*v*) Tween 80 at 37 °C. Data represents mean ± SD (n = 3).

In vitro drug release from PACAlg-CAB microspheres was studied over an 8-day period (Figure 4, purple line). The results showed a gradual drug release during the first two days of incubation, before the curve flattened. The estimated release rate constant was 0.27 (95% Confidence Interval (CI): 0.140–0.406) h^−1^ with a maximum release of 67%. To gain insight into the role of the NP/alginate gel matrix in drug release kinetics, alginate microspheres were loaded with free CAB with or without empty PACA NPs (Figure 4, red and blue lines, respectively). The results showed that 92% of the CAB content was momentarily released from the microspheres when free CAB was encapsulated alone. Exponential modeling of drug release was not possible, and a rate constant could therefore not be estimated. Co-encapsulation of free CAB with empty NPs showed a slight restriction of CAB release, with an 81% drug release after 1 h of incubation and a final plateau at 75% after 2 days. This gave a release rate constant of 2.90 (95% CI: 2.02–3.74) h^−1^—more than 10 times higher and statistically different from that of PACAlg-CAB microspheres. 

### 2.4. In Vitro Cytotoxicity Studies 

**Figure 5:**
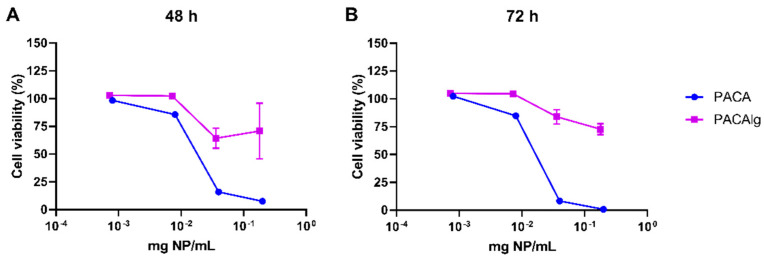
Cytotoxicity profile of PACAlg in LP-9 mesothelial cells. Cell viability of LP-9 cells after (**A**) 48 h and (**B**) 72 h of incubation with empty PACA NPs (blue lines) or PACAlg microspheres (purple lines). Data represents mean ± SD (n = 4).

The human mesothelial cell line LP-9 was used to evaluate the cytotoxicity of empty PACA NPs and alginate-encapsulated PACA NPs. Figure 5 shows that alginate encapsulation limits the reduction in cellular viability after both 48 h (Figure 5A) and 72 h incubation (Figure 5B) and especially at high NP concentrations. 

### 2.5. In Vivo Biodistribution 

**Figure 6:**
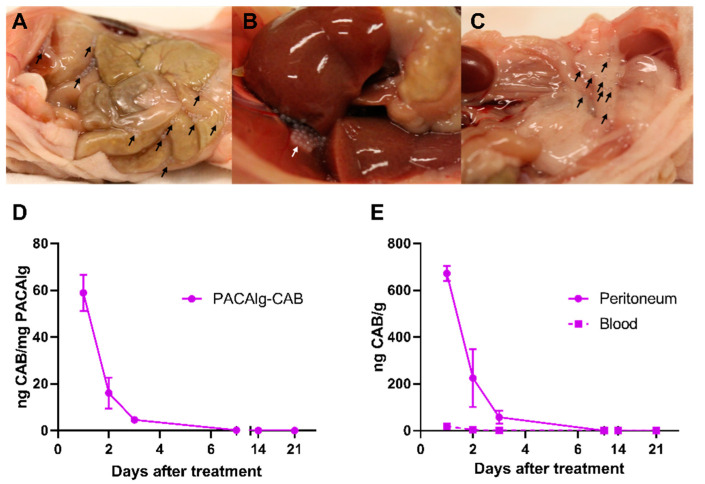
In vivo biodistribution of PACAlg-CAB microspheres. Representative images showing the distribution of PACAlg-CAB microspheres (**A**) between the intestines, (**B**) accumulation between the liver and diaphragm, and (**C**) in adipose tissue following intraperitoneal administration in healthy mice. Arrows indicate visible microspheres. (**D**) Remaining concentrations of CAB in PACAlg-CAB microspheres after intraperitoneal administration of PACAlg-CAB (6 mg CAB/kg). (**E**) CAB concentrations in peritoneum and blood after intraperitoneal administration of PACAlg-CAB (6 mg CAB/kg). Data show the mean value with spread indicating the two individual values per timepoint.

PACAlg-CAB microspheres were intraperitoneally injected in healthy mice, and blood, liver, spleen, peritoneum, adipose tissues, and PACAlg-CAB were harvested at 1 to 21 days after administration. At all the examined timepoints, the microspheres were found to be intact and distributed throughout the abdominal cavity, including between the intestines, between the liver and the diaphragm and in the adipose tissues in the pelvis, mesentery and omentum (Figure 6A–C). Extraction of encapsulated CAB from PACAlg-CAB microspheres showed that most of the CAB content was released during the first 3 days after implantation, followed by a slower release up to day 7 (Figure 6D). The average measured concentrations remaining within the microspheres after 1, 2, and 3 days corresponded to 2.9%, 0.80%, and 0.23% of the initial theoretical maximum value, respectively. CAB measurement of extracted blood and a representative piece of peritoneal tissue showed an enrichment of the drug in the peritoneum compared to the systemic circulation (Figure 6E). Complete drug clearance was seen in the blood, liver, and spleen after 3 days, and after 7 days for the intraperitoneal tissues (peritoneum and adipose tissue) (Supplementary Figure S1). 

### 2.6. Treatment Efficacy 

**Figure 7:**
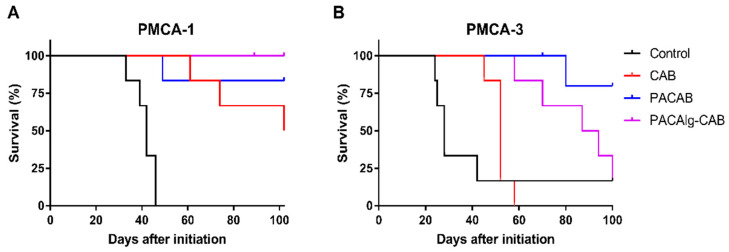
Treatment efficacy of PACAlg-CAB microspheres. Kaplan Meier curves showing mice treated with saline (control), CAB, PACAB, or PACAlg-CAB (15 mg CAB/kg) in the PDX models (**A**) PMCA-1 and (**B**) PMCA-3 (n = 5–6 mice per group).

To investigate the treatment efficacy of PACAlg-CAB microspheres in vivo, mice injected with the mucinous PDX models PMCA-1 or PMCA-3 were treated with saline control, CAB, PACAB, or PACAlg-CAB for survival evaluation (Figure 7). The PMCA-1 model responded strongly to all treatments involving CAB, in which 3/6, 5/6, and 5/5 mice were considered cured with no visible tumor after treatment with CAB, PACAB, and PACAlg-CAB, respectively (Figure 7A). No statistically significant differences were observed between the treatment groups. For PMCA-3, treatment with PACAB or PACAlg-CAB significantly increased the survival in mice compared to mice treated with free CAB, and 2/5 and 1/6 mice were cured after treatment with PACAB and PACAlg-CAB, respectively (Figure 7B). There was no significant difference between the PACAB and the PACAlg-CAB group. The p-values between the treatment groups are shown in Supplementary Table S1. 

## 3. Discussion

Peritoneal metastases are associated with poor prognosis, and more efficacious treatment options are needed [10,11]. Drug encapsulation in NPs has previously shown promising effects by delaying systemic drug absorption through prolongation of the peritoneal residence time [13,21,22]. Due to their physicochemical characteristics, NPs are less prone to clearance from the peritoneal cavity, and the increased retention leads to higher local drug concentrations that can result in improved treatment efficacy. In this study, novel biocompatible alginate microspheres encapsulating drug-loaded NPs (PACAlg-CAB) were developed with the aim to further increase peritoneal retention and reduce toxic side effects, and hence improving cancer treatment efficacy of cytostatic drugs. To our knowledge, this is the first time alginate has been used as a microparticulate carrier to encapsulate NPs for the treatment of intraperitoneal cancer. 

Electrostatic droplet generation was used to synthesize spherical and monodisperse PACAlg alginate hydrogels with an average diameter of approximately 450 µm. The microspheres were shown to be stable with respect to size and morphology for at least 8 days in physiological conditions in vitro. Fluorescence stability over the same period indicated that the alginate forms rigid and stable crosslinked networks that retain the fluorescently labeled NPs within the microspheres, even after initial osmotic swelling. This shows that NP degradation is needed in order to release the active pharmaceutical ingredient (API) from the microspheres. The in vivo biodistribution study gave similar results, showing microsphere stability for at least 21 days after injection in the peritoneal cavity in mice. This demonstrates that alginate forms crosslinked hydrogel networks that are stable also at in vivo conditions. 

Drug release data showed that the alginate microspheres alone have limited retention capacities on the CAB drug. CAB is a relatively small hydrophobic drug (836 Da), which is not expected to interact with the highly hydrophilic alginate backbone or be retained in the alginate porous network [23]. Co-encapsulation with empty NPs showed a slight restriction of CAB release, suggesting an increased interaction between CAB and the NP/hydrogel matrix compared to that of CAB and the pure alginate gel. This was not expected, as the hydrophilic surfaces of PEGylated NPs were thought to have a minimal affinity towards CAB. The observed retention could therefore be a consequence of NP degradation, with CAB showing affinity to hydrophobic components of the PACA polymers or their degradation products. This may also explain the lack of complete (100%) drug release seen in vitro for both the PACAlg-CAB (67%) and PACAlg with co-encapsulated CAB (75%) samples.

PACAlg-CAB drug release data showed a gradual drug release over the first two days in vitro, before the curve reached the plateau. The release rate could possibly be further prolonged by using NPs with an inherently longer drug release rate, such as a PACA polymer with longer alkyl side-chains [24]. PACAlg drug release could also be tuned by editing the chemical composition of the alginate hydrogel (the secondary barrier) by introducing polymers or polymer side chains with a varying affinity towards the API. It was previously shown that cyclodextrin-grafted alginates can modulate the drug release of paclitaxel [25]—a secondary barrier that potentially also could be applied to the PACAlg drug delivery system. 

As shown by the fluorescence stability study, NPs were not released from the microspheres within the observation period. One can thereby assume drug release as a free drug only—firstly as a release from the PACA NPs, secondly as diffusion out of the alginate network. This eliminates cellular uptake of NP-encapsulated drugs, and leaves diffusion of the hydrophobic drug across the cellular membranes as the most probable drug delivery mechanism. 

Even though NP-based drug encapsulation for peritoneal administration is an appealing strategy, it may involve toxic effects, including NPs entering the systemic circulation, accumulation of NPs in the spleen and liver, and inflammatory responses in the peritoneum [26]. NPs of various sizes and materials have also been shown to cause ascites production in tumor-bearing mice, resulting in reduced survival [27]. New strategies to reduce the toxic effects are therefore needed, and it was hypothesized that NP encapsulation in alginate microspheres could reduce the toxic potential of intraperitoneal NPs. Alginate has commonly been explored for intraperitoneal administration, e.g., for encapsulation of insulin-producing islet cells and hepatocytes [28,29]. The in vitro cytotoxicity results presented in the current study show promising effects of alginate encapsulation. The alginate hydrogel was non-toxic for normal mesothelial cells (LP-9), and encapsulation reduced the toxic effects of the NP material. Mesothelial cells line the surface of the peritoneal cavity and are therefore a highly relevant cell model for toxicity evaluation of novel intraperitoneal treatment strategies.

In this study, the taxane CAB was used as the API for cancer therapy. CAB is a highly potent chemotherapeutic agent with the drawback of inducing severe toxic side effects in healthy tissues [15]. Encapsulation of CAB into nanocarriers is shown to reduce these side effects, and also prolong blood circulation half-lives [30]—allowing for efficient preclinical antitumor effects after dose regimens of 6–15 mg/kg in mice [18,19]. For APIs where high cumulative doses are required for treatment effect, e.g., doxorubicin [31], a higher number of NPs would be needed to deliver the necessary doses. The observed reduction of NP-based toxic side effects by alginate encapsulation could therefore be essential to allow the administration of adequate doses of other APIs, thereby allowing novel treatment strategies for these drugs.

Peritoneal metastases may be located throughout the entire peritoneal cavity, representing a large surface area, as the peritoneal area is equivalent to the body surface area in humans [32,33]. To achieve optimal treatment efficacy, it is therefore important that the cytotoxic drugs are distributed throughout the peritoneal cavity to ensure drug exposure to all metastases. PACAlg-CAB microspheres were shown to be well distributed throughout the peritoneal cavity, without adhesion to tissue surfaces. The therapeutic potential of PACAlg-CAB was evaluated in two relevant PDX models mimicking peritoneal metastasis. The PMCA-1 model was highly CAB sensitive, and no significant improvement in survival was observed by any of the NP-based delivery systems. Interestingly, however, all PACAlg-CAB-treated mice were cured by the treatment, while for the other groups some mice eventually had to be sacrificed because of peritoneal metastases. In the PMCA-3 model, both PACAB and PACAlg-CAB significantly improved survival compared to CAB alone. This is a high-grade tumor with signet cell differentiation, which is known for being an aggressive phenotype that is hard to treat. The in vivo results clearly show that the treatment efficacy of CAB was at least maintained by NP encapsulation. However, incorporating PACAB into the alginate microspheres did not further increase drug efficacy, and for CAB, in particular, the additional retention and reduced toxicity may not be necessary. However, for drugs with high unspecific toxicity that must be delivered at high doses, for drugs where the NP loading capacity is lower than for CAB, where high amounts of NP must be delivered, the PACAlg drug delivery platform could still represent an important improvement. 

In conclusion, this proof-of-concept study presents the alginate encapsulation of drug-loaded NPs as a promising novel strategy for drug administration in the peritoneal cavity that could improve the therapeutic ratio of toxic drugs. PACAlg allows for reduced cytotoxicity, the possibility of administering higher drug doses, and an increased ability to tune drug release rates compared to that of NPs alone. The technology could be further improved by exchanging the API or by tuning the drug release rate by chemical modification of the alginate hydrogel. 

## 4. Materials and Methods

### 4.1. Synthesis and Characterization of PACA Nanoparticles 

PACA NPs were synthesized using the miniemulsion polymerization technique, as previously described [19,34,35]. Briefly, oil-in water-emulsions were prepared by mixing a monomer oil phase with an acidic water phase. The oil phase contained ethylbutyl-cyanoacrylate (EBCA; Cuantum Medical Cosmetics, Bellaterra, Spain), the co-stabilizer MIGLYOL^®^ 812 N (2.3% (*w*/*w*), Cremer OLEO GmbH & Co. KG, Hamburg, Germany), and the payload (0.2% (*w*/*w*) of the fluorescent dye NR668 (modified Nile Red, custom synthesis [36])) and/or the cytostatic agent CAB (10.2% (*w*/*w*); Biochempartner Ltd., Wuhan, China)). The water phase consisted of the non-ionic PEG stabilizers Brij^®^ L23 (6.8 mM, Sigma-Aldrich, St. Louis, MO, USA) and Kolliphor^®^ HS 15 (8.7 mM in 0.1 M HCl; Sigma-Aldrich (St. Louis, MO, USA)). The emulsions were sonicated (50% amplitude, 450 Digital Sonifier^®^, Branson Ultrasonics, Danbury, CT, USA) in an ice bath for 3 min, and subsequently stirred overnight at room temperature. The pH was adjusted to 5 with 0.1 M NaOH, and the stirring was continued for at least 5 h. Dialysis (Spectra/Por dialysis membrane, 12–14 kDa MWCO; Spectrum Labs, Rancho Dominguez, CA, USA) against 1 mM HCl was performed to remove any excess stabilizers from the NP solutions. 

The synthesized NPs were characterized for size (Z-average), polydispersity index (PDI), and surface charge (zeta potential) using electrophoretic and dynamic light scattering (ELS and DLS) (Zetasizer Nano ZS, Malvern Instruments/Malvern Panalytical, Malvern, UK) in 0.01 M phosphate buffer, pH 7. 

### 4.2. Synthesis and Characterization of PACAlg Microspheres 

PACAlg microspheres were produced by an extrusion-dripping technique using an electrostatic bead generator (the prototype designed by the Trondheim Bioencapsulation Group, NTNU, and the commercially available version from Nisco, Zurich, Switzerland) [37]. First, a sodium alginate solution (2.2% (*w*/*v*) PRONOVA™ UP LVG, pH 5; NovaMatrix, Sandvika, Norway) was mixed with the PACA NP solution to form a suspension with alginate concentration of 1.8% (*w*/*v*) and 18% (*v*/*v*) NP stock solution. The suspension was stirred overnight at 4 °C to form a homogeneous suspension and to release air bubbles. Microspheres were synthesized by extruding the alginate solution through a nozzle (350 µm outer diameter, 170 µm inner diameter) by using a syringe pump (Cole-Parmer, Vernon Hills, IL, USA) at a flow rate of 10 mL/h, an applied voltage of 7.0 kV and a 4.5 cm collecting distance down to a calcium chloride (50 mM, pH 5; Sigma-Aldrich (St. Louis, MO, USA), hardening bath where the microspheres were left to crosslink for 10 min at room temperature under magnetic stirring. Empty microspheres, microspheres encapsulating free drug, and microspheres co-encapsulating free drug together with empty NPs were synthesized as controls. 

The size and shape of the microspheres were characterized by phase contrast and fluorescence microscopy (Nikon Eclipse TS100 with a Plan Flour 4X/0.13 NA objective with a pE-4000 CoolLED illumination system and a TRITC Quad filter set, λ_ex_ = 550 nm; Nikon, Tokyo, Japan). ImageJ2 [38] was used to determine the average diameter of 40 microspheres imaged by phase contrast. *Aspect ratio (AR)* and *sphericity factor (SF)* were calculated for the same microspheres by using the Analyze particles function in ImageJ2 and the following equations:

$$Aspect\;ratio\;\left(AR\right)=\frac{D_{Max}}{D_{Orthogonal}}$$

$$Sphericity\;factor\;\left(SF\right)=\frac{D_{Max}-D_{Min}}{D_{Max}+D_{Min}}$$

where *D_Max_* is the maximum Feret diameter, *D_Orthogonal_* is the orthogonal diameter and *D_Min_* is the minimum Feret diameter [39]. Microspheres with *AR* < 1.1 and *SF* < 0.05 were considered spherical. Swelling behavior and size stability were studied by incubating microspheres in PBS with calcium chloride and magnesium chloride (1:450 dilution factor; D1283, Sigma-Aldrich (St. Louis, MO, USA), pH 7.4 at 37 °C in the dark. 

The NP distribution in the alginate microspheres was examined by confocal laser scanning microscopy (CLSM), using a Leica SP8 microscope with a 25X/0.95 NA water objective and a 20X/0.75 NA dry immersion objective (Leica Microsystems, Wetzlar, Germany). The NP-encapsulated fluorophore NR668 was excited by a white light laser at 534 nm, and emission was detected at 574–627 nm. 

The retention of NPs within the alginate microspheres was quantified by measuring the fluorescence intensity of microspheres filled with dye-loaded NPs. The microspheres were diluted (1:450 volumetric dilution factor) in PBS with calcium chloride and magnesium chloride, pH 7.4, at 37 °C in the dark, and analyzed on a microplate reader (SpectraMax i3x, Molecular Devices, San Jose, CA, USA, λ_ex_ = 514 nm, λ_em_ = 600 nm). The retention of NPs was calculated as follows:

$$Retention\;\left(\%\right)=\frac{F_{Initial}-F_{Measured}}{F_{Initial}}\times 100\%$$

where *F_Initial_* is the initial fluorescence intensity of the incubated microspheres and *F_Measured_* is the fluorescence intensity of the medium at specific time points. 

Drug release from the microspheres was examined by incubating microspheres loaded with PACAB in PBS with calcium chloride and magnesium chloride (1:450 dilution factor), pH 7.4 and supplemented with 0.5% (*w*/*v*) Tween 80 to ensure drug solubility. Aliquots (100 µL) were withdrawn at predetermined time points, diluted 1:100 in acetone, and analyzed for drug content by liquid chromatography tandem mass spectrometry (LC-MS/MS) as described below. The experiments were performed in independent triplicates, and conducted at 37 °C in the dark under mild stirring for up to 8 days. Two control groups: I: microspheres encapsulating free drug and II: free drug mixed with microspheres encapsulating empty NPs were included in the experiment. Percentage drug release was determined using the following equation:

$$Drug\;release\;\left(\%\right)=\frac{C_{Measured}}{C_{Max}}\times 100\%$$

where *C_Max_* is estimated by:

$$C_{Max}=\frac{C_{Alg\;}\times V_{Alg}-\left(C_{Gel}\times V_{Gel}\right)}{V_{Medium}}$$

where *C_Alg_* and *V_Alg_* are the calculated drug concentration and volume of the alginate solution used for encapsulation, respectively. *C_Gel_* and *V_Gel_* are the quantified drug concentration and volume of the alginate gelling solution, respectively. *V_Medium_* denotes the volume of the PBS incubation medium. The release data was fitted to an exponential release model with a nonlinear least square approach, using the Curve Fitting Toolbox in Matlab (Release 2018a, The MatWorks, Inc., Natick, MA, USA). Data was fitted to the following equation:

$$Release\;\left(t\right)=\;K\left(1\;-\;e^{-kt}\right)\;+\;K_{0}$$

where Release (*t*) is the percentage release at time *t* after microsphere incubation, *K*_0_ the instantaneous release at *t* = 0, k the release rate constant and *K* + *K*_0_ the steady-state conditions. 

### 4.3. CAB Quantification by LC-MS/MS 

CAB content was quantified by LC-MS/MS as previously described [18], using an Agilent 1290 HPLC system coupled to an Agilent 6495 triple quadrupole mass spectrometer, equipped with an Agilent Jet Stream ion source (Agilent Technologies, Santa Clara, CA, USA). CAB was chromatographically separated from the matrix using an Ascentis^®^ Express C8 column (75 × 2.1 mm, 2.7 μm particle size), with a 5 × 2.1 mm guard column of the same material (Sigma-Aldrich, St. Louis, MO, USA), at 40 °C. Mobile phase A was 25 mM formic acid in water and Mobile phase B was 100% methanol, run at a flow rate of 0.5 mL/min. The mobile phase gradient was isocratic at 55% B for 1.5 min, then increased to 80% B over 1 minute, followed by 1 min washout time and subsequently column re-equilibration. This resulted in a retention time of 3.1 min and a run time of 5.0 min per injection. Injection volume was 5 μL. MS detection was in positive mode, quantified in multiple reaction monitoring (MRM) mode using the transition *m/z* 858.3 → 577.2. The parent ion was chosen to be the Na adduct as this gave the best sensitivity. Similarly, the hexadeuterated internal standard was detected on the 864.4 → 583.2 transition. Both analytes were run at 380 V fragmentor and 20 V collision energy voltages. 

Reference standards were used to ensure accurate quantification. The unlabelled CAB standard used for NP encapsulation was dissolved in acetone to 1 mg/mL and used to generate a standard curve spanning at least five concentration points. The limit of quantification (LOQ) was 1 ng/mL. The hexadeuterated internal standard (Toronto Research Chemicals Inc, Toronto, Canada; catalogue number C046502, 99.6% isotopic purity) was dissolved to 1 mg/mL in acetone and added at the same concentration to all standards and samples to compensate for possible matrix effects.

CAB encapsulation efficiency (%) in PACA NPs was calculated by the following formula:

$$Encapsulation\;efficiency\;\left(\%\right)=\frac{Drug\;amount\;added\;during\;synthesis}{Drug\;amount\;encapsulated}\times 100\%\;$$

### 4.4. In Vitro Cytotoxicity Studies 

LP-9 normal human mesothelial cells (AG07086, Coriell Institute for Medical Research, Camden, NJ, USA) were cultivated in a humidified atmosphere at 5% CO_2_ and 37 °C in Medium 199 (M2154, Sigma-Aldrich (St. Louis, MO, USA)/MCDB110 (mixed 1 + 1). The MCDB110 medium was prepared in-house as follows: MCDB110 powder (15.3 g, US Biological, Salem, MA, USA) and sodium bicarbonate (2.2 g, Sigma-Aldrich, St. Louis, MO, USA) were dissolved in 900 mL distilled water. pH was adjusted to 7.4 by adding 2 M NaOH before the volume was brought to 1 L with distilled water. The solution was sterile filtered before use. The Medium 199/MCDB110 solution was supplemented with 1% (*v*/*v*) L-glutamine (G7513, Sigma-Aldrich, St. Louis, MO, USA), 1% (*v*/*v*) penicillin-streptomycin (15140122, Thermo Fisher Scientific, Waltham, MA, USA), 15% (*v*/*v*) fetal bovine serum (F7524, Sigma Aldrich, St. Louis, MO, USA), 10 ng/mL epidermal growth factor (E9644, Sigma-Aldrich, St. Louis, MO, USA) and 0.4 µg/mL hydrocortisone (H0888, Sigma-Aldrich, St. Louis, MO, USA). 

For toxicity screening, LP-9 cells were seeded by adding 1875 cells (100 µL) per well in 96 well plates (165305, Thermo Fisher Scientific, Waltham, MA, USA). Following 24 h of incubation, empty PACA NPs or PACAlg were added at concentrations ranging from 0.0008 to 2 mg NPs/mL or 0.00072 to 0.18 mg NPs/mL, respectively. Staurosporine (S4400, Sigma-Aldrich, St. Louis, MO, USA) was included as positive control (0.0016 to 0.042 mg/mL). The plates were incubated for 48 or 72 h before viability was assessed by the addition of CellTiter-Glo 2.0 assay (Promega, Madison, WI, USA) (1 + 1). The luminescence signal was detected with a SpectraMax i3x multi-mode microplate reader after 10 min.

### 4.5. In Vivo Studies 

All procedures and experiments involving mice were approved by the Norwegian Food Safety Authority (application ID #18209) and conducted according to the recommendations of the European Laboratory Animals Science Association and the ARRIVE guidelines [40]. Female athymic nude foxn1^nu^ mice (6–8 weeks, 20–25g) were bred at the Department of Comparative Medicine, The Norwegian Radium Hospital, and kept in a specific pathogen free environment at constant temperature (22 ± 1 °C) and humidity (62 ± 5%) and with 15 air changes/hour and a 12-h light/dark cycle. Mice were purchased at 3 weeks, and then moved from the breeding room to the experimental room and allowed to acclimatize for 3–5 weeks until the start of the experiments. A maximum of nine mice were housed in each cage. Food and water were supplied ad libitum, and mice were given cardboard houses and paper to have nesting material and for environmental stimulation, as required by the Department of Comparative Medicine at the Norwegian Radium Hospital to improve the welfare of the mice [41]. 

### 4.6. Biodistribution Study

For biodistribution experiments, PACAlg-CAB (6 mg CAB/kg, 500–600 µL) was injected intraperitoneally. Mice were euthanized after 1, 2, 3, 7, 14, and 21 days (two mice per time point) by cervical dislocation after a cardiac puncture to collect blood under 3% sevofluran anesthesia. Liver, spleen, peritoneum, adipose tissue, and a section of the parietal peritoneum, were excised from near the injection site and visible PACAlg-CAB microparticles were harvested. All samples were snap-frozen in liquid nitrogen and stored at −80 °C until further processing and LC-MS/MS analysis as described above. Blood was collected in EDTA tubes (Sarstedt Microvette^®^ K3 EDTA, 500 µL, Sarstedt, Nümbrecht, Germany), mixed, and stored at −80 °C before LC-MS/MS analysis. 

The tissue homogenization protocol published by Fusser, et al. 2019 [18] was used to process the sample tissues and release the CAB content. A total of 1 mL of freshly prepared enzyme solution was added per 50 mg tissue and incubated at 37 °C for 48 h until the tissue was completely dissolved. Drug stability under these conditions was verified by including a free CAB control. All tissue homogenates and blood samples were diluted 10× in acetone added internal standard to extract the free drug and simultaneously precipitate sample proteins. The protein precipitates were then sedimented by centrifugation, and the supernatant was transferred to an HPLC vial for analysis. PACAlg-CAB microparticles were accurately weighed and transferred to Eppendorf tubes with 100 µL acetone added internal standard. All tubes were left for drug extraction by gentle rotation on a rotating mixer for 48 h at room temperature. The microparticles were then sedimented by centrifugation, and the supernatant was transferred to vials for analysis.

### 4.7. Treatment Efficacy Study

For treatment efficacy experiments, the PDX models PMCA-1 and PMCA-3 were used. The establishment of the models from patients with mucinous peritoneal metastases was previously described [42]. Both models were established by implanting peritoneal tissue samples collected at the time of CRS-HIPEC. PMCA-1 was derived from a patient with a primary rectal carcinoma, while PMCA-3 was derived from a patient with high-grade mucinous cancer with signet cell differentiation. 200 µL of mucinous ascites were injected intraperitoneally. Treatment with 15 mg/kg CAB, PACAB, or PACAlg-CAB (500–600 µL per mouse) was initiated the following day to mimic the clinical situation after CRS with a low tumor load intraabdominally. CAB was dissolved to 40 mg/mL in Tween-80 (Sigma-Aldrich, St. Louis, MO, USA), and then in 10 mg/mL in 13% ethanol, before diluting in 0.9% NaCl to achieve the correct concentration. PACAB and PACAlg-CAB were diluted in 0.9% NaCl. Mice were randomly distributed to treatment groups of six mice. The mice were euthanized by cervical dislocation when they displayed a large distended abdomen or 100–102 days after treatment initiation if the mice did not develop a tumor. Two mice (PMCA-1, PACAlg-CAB, and PMCA-3, PACAB) were excluded from analyses due to death not occurring from tumor growth. 

### 4.8. Statistical Analysis 

Statistical analyses were performed using GraphPad Prism v7 and v9 (GraphPad Software, LaJolla, CA, USA). Student’s *t*-tests were used to compare differences between treatment groups. Survival curves (Kaplan–Meier plot) were compared using the Gehan-Breslow Wilcoxon test. *p* values < 0.05 were considered significant.

## 5. Patents

Ý.M. has patent #WO 2019/185685 pending to SINTEF TTO. Ý.M., K.F. and K.G.F. have patent #WO 2020/192950 and patent #US 16/366596 pending to SINTEF TTO.

## Data Availability

Not applicable.

## Supplementary Materials

The following supporting information can be downloaded at: https://www.mdpi.com/article/10.3390/md20120744/s1, Figure S1: Biodistribution of CAB in liver, spleen and adipose tissue after intraperitoneal administration of PACAlg-CAB. Supplementary Table S1: *p*-values for treatment efficacy study.
