# Supplementary material for: Alginate Microsphere Encapsulation of Drug-Loaded Nanoparticles: A Novel Strategy for Intraperitoneal Drug Delivery
Source: Mar Drugs. 2022 Nov 26;20(12):744. doi: 10.3390/md20120744 (PMC9782800; doi:10.3390/md20120744)
Supplement: Supplementary file 1 [file marinedrugs-20-00744-s001.zip › marinedrugs-2034949-supplementary.docx]

Supplementary information

Scheme 1. Biodistribution of CAB in liver, spleen and adipose tissues after intraperitoneal administration of PACAlg-CAB (6 mg/kg) in healthy mice. Data shows the mean value with spread indicating the two individual values per timepoint.

Supplementary Table 1. Overview of the p-values between the different treatment groups in the treatment efficacy studies. Significance was calculated using Gehen-Beslow Wilcoxen test. Significant differences are marked in bold.

**PMCA-1**

|  | **CAB** | **PACAB** | **PACAlg-CAB** |
| --- | --- | --- | --- |
| **Control** | **0.002** | **0.002** | **0.002** |
| **CAB** |  | 0.390 | 0.067 |
| **PACAB** |  |  | 0.317 |

**PMCA-3**

|  | **CAB** | **PACAB** | **PACAlg-CAB** |
| --- | --- | --- | --- |
| **Control** | **0.039** | **0.010** | **0.035** |
| **CAB** |  | **0.001** | **0.002** |
| **PACAB** |  |  | 0.070 |
